# Tailored design of NKT-stimulatory glycolipids for polarization of immune responses

**DOI:** 10.1186/s12929-017-0325-0

**Published:** 2017-03-23

**Authors:** Jung-Tung Hung, Jing-Rong Huang, Alice L. Yu

**Affiliations:** 1 Institute of Stem Cell & Translational Cancer Research, Chang Gung Memorial Hospital at Linkou and Chang Gung University, No. 5, Fu-Shin St., Kuei Shang, Taoyuan, 333 Taiwan; 2 Department of Pediatrics, University of California in San Diego, San Diego, CA USA

**Keywords:** iNKT cell, α-galactosylceramide, Anergy, Myeloid-derived suppressive cell

## Abstract

Natural killer T (NKT) cell is a distinct population of T lymphocytes that can rapidly release massive amount of Th1 and Th2 cytokines upon the engagement of their T cell receptor with glycolipids presented by CD1d. The secreted cytokines can promote cell-mediated immunity to kill tumor cells and intracellular pathogens, or suppress autoreactive immune cells in autoimmune diseases. Thus, NKT cell is an attractive target for developing new therapeutics to manipulate immune system. The best-known glycolipid to activate NKT cells is α-galactosylceramide (α-GalCer), which has been used as a prototype for designing new NKT stimulatory glycolipids. Many analogues have been generated by modification of the galactosyl moiety, the acyl chain or the phytosphingosine chain of α-GalCer. Some of the analogues showed greater abilities than α-GalCer in polarizing immune responses toward Th1 or Th2 dominance. Among them, several analogues containing phenyl groups in the lipid tails were more potent in inducing Th1-skewed cytokines and exhibited greater anticancer efficacy than α-GalCer. Analyses of the correlation between structure and activity of various α-GalCer analogues on the activation of iNKT cell revealed that CD1d–glycolipid complexes interacted with the same population of iNKT cell expressing similar T-cell receptor Vβ as α-GalCer. On the other hand, those phenyl glycolipids with propensity for Th1 dominant responses showed greater binding avidity and stability than α-GalCer for iNKT T-cell receptor when complexed with CD1d. Thus, it is the avidity and stability of the ternary complexes of CD1d-glycolipid-iNKT TCR that dictate the polarity and potency of immune responses. These findings provide a key to the rationale design of immune modulating glycolipids with desirable Th1/Th2 polarity for clinical application. In addition, elucidation of α-GalCer-induced anergy, liver damage and accumulation of myeloid derived suppressor cells has offered explanation for its lacklustre anti-cancer activities in clinical trials. On other hand, the lack of such drawbacks in glycolipid analogues containing phenyl groups in the lipid tails of α-GalCer coupled with the greater binding avidity and stability of CD1d-glycolipid complex for iNKT T-cell receptor, account for their superior anti-cancer efficacy in tumor bearing mice. Further clinical development of these phenyl glycolipids is warranted.

## Background

Natural killer T (NKT) cells play a central role in connecting innate immunity and adaptive immunity. They can modulate immune responses by orchestrating other immune cells, including T cells, B cells, natural killer (NK) cells and dendritic cells (DCs) [1]. There are two major subpopulations of NKT cells, classical (or type I) NKT cells and non-classical (or type II) NKT cells [2–4]. Those two subpopulations of NKT cells are response to lipid antigens in a CD1d-dependent manner. Many glycolipid antigens for NKT cells have been identified, including bacterial glycolipids such as α-galacturonosyl ceramide, α-glucuronosyl ceramide and α-galactosyl diacylglycerol and mammalian glycolipids such as isoglobotrihexosylceramide (iGb3) and disialoganglioside GD3 [5–7]. In addition, α-galactosylceramide (α-GalCer) isolated from marine sponge is found to have a potent activity to activate the classical NKT cells and display anticancer effects in tumor-bearing mouse model. The robust immune stimulating activities of α-GalCer inspire researchers to use the α-GalCer as template to design more potent immune modulating glycolipids. For example, glycolipid OCH prevents the experimental autoimmune encephalomyelitis [8], α-C-Gal protects mice against malaria and melanoma metastases [9] and phenyl-glycolipids suppress tumor growth in mouse models as well as strong adjuvant effect on DNA vaccine [10, 11]. The differential biological activities of various α-GalCer analogues provide valuable insights into the relationship between structural modification of α-GalCer and the immune-modulating activities and thereby facilitating the design of novel analogues with desirable properties for various clinical applications.

## Subsets and functions of NKT cells

NKT cells are a unique subset of T lymphocytes that coexpress α/β T cell receptor (TCR) and NK lineage markers, i.e. NK1.1, CD122 (IL-2Rβ) and various Ly49 molecules. Based on the phenotype and content of cytokines, NKT cells have been divided into two main subsets: type I NKT cells, known as invariant NKT (iNKT) cells producing IFN-γ, IL-2, IL-4, IL-5 and IL-13, and type II NKT cells which encompass many diverse NKT cells secreting IFN-γ, TNF-α, IL-17A and IL-6 [12]. The iNKT cells can be found in thymus, liver, bone marrow, spleen and peripheral blood. In mice, iNKT cells comprise of approximately 1 to 3% of lymphocytes in the circulation and are enriched in the liver where iNKT can constitute up to 30% of resident lymphocytes. These cells are CD1d-restricted CD4^+^ or CD4^−^CD8^−^ T cells with NK markers and exhibit an activated phenotype (CD44^high^Ly6C^high^ IL-2Rβ^high^). The TCR usage of iNKT cells is quite unique with a semi-invariant α-chain consisting of Vα14Jα18, and preferential usages of Vβ2, Vβ7 or Vβ8.2 for β-chain in mice [13–18]. In human, a similar population of cells expressing Vα24Jα18 and Vβ11 has been identified [19, 20]. Although the type II NKT cells are also responsive to CD1d-presented glycolipids, which do not include α-GalCer [21], they express polyclonal TCR repertoires similar to the highly diverse TCRs of conventional CD4 and CD8 T cells.

Unlike conventional T cells, which recognize peptides presented by major histocompatibility complex (MHC) molecules, both iNKT or type II NKT cells recognize glycolipid antigens presented by CD1d proteins which are nonpolymorphic MHC class I–like molecules [22–24]. CD1d proteins are expressed on cells of hematopoietic origin such as dendritic cells, B cells, T cells and macrophages [25]. According to the crystal structure of CD1d protein, antigen-binding site of CD1d molecules is composed of two channels, A’ and F’ channels, which bind to an acyl chain and a phytosphingosine chain, respectively [26–31]. Antigens such as glycosylceramide and glycosylphosphatidylinositol could be presented by CD1d. Their alkyl chains are inserted into hydrophobic grooves A’ and F’ channels of CD1d so that their carbohydrate moieties protrude to contact with TCR of NKT cells [23, 32].

Upon engagement with CD1d/α-GalCer complex, iNKT cells become activated with rapid production of cytokines within minutes [33], such as interleukin-4 (IL-4) and interferon-γ (IFN-γ), along with upregulation of activation markers such as CD69, augmented cell proliferation and increased cytotoxic capacity [23, 34–36]. The secreted cytokines will not only trigger activation of T cells, NK cells, B cells and dendritic cells but also direct immune responses toward Th1 or Th2 responses [37, 38]. For instance, IFN-γ promotes Th1 cell differentiation and NK cell activation, which is essential for defense against tumors and various intracellular pathogens. Conversely, IL-4 controls the initiation of Th2 responses, which inhibit Th1-mediated autoimmune responses such as collagen-induced arthritis (CIA), experimental autoimmune encephalomyelitis (EAE) and type I diabetes in NOD mice. Hence, iNKT cells are thought to play a central role in innate and adaptive immunity against viruses, bacteria, parasites, autoimmune diseases and cancer [23, 39–42].

Type II NKT cells do not respond to α-GalCer and therefore they cannot be identified by α-GalCer/CD1d tetramers. Such technical limitations have hindered efforts to interrogate type II NKT cells and, consequently, relatively little is known about their roles in immune system. So far, a major subset of type II NKT cells has been found to respond to β-linked self-glycolipid sulfatide [43]. Comparing the crystal structures of type I NKT-α-GalCer/CD1d complex and type II NKT cell TCR-sulfatide/CD1d complex, Girardi et al. found that the type II NKT TCR bound to sulfatide/CD1d with an perpendicular orientation but the type I NKT TCR bound to α-GalCer/CD1d with an diagonal orientation [44]. This study suggests that the immune-modulating effects of iNKT and type II NKT cells might be quite different. Indeed, it is known that type II NKT cells played an important role in anergy induction in the inflammatory liver [45], suppression of graft-versus-host disease (GVHD) [46] and inhibition of airway asthma induced by Type I NKT cells in mouse model [47]. In addition, type II NKT cells might be involved in cancer progression by secreting IL-13 to inhibit tumor-specific CD8^+^ T cells [48]. In human, an increase in IL-13-secreting type II NKT cells stimulated by lysophosphotidylcholine was observed in patients with multiple myeloma [49]. Moreover, tumor growth was significantly increased in Jα18^−/−^ mice, which lack type I NKT cells but still retain type II NKT cells, compared to that in CD1d^−/−^ mice, which lack both type I and type II NKT cells [50]. Similarly, Izhak et al. demonstrated that enhanced tumor growth in Jα18^-/-^ mice was not undermined by blocking regulatory T (Treg) cell blockade with anti-CD25 antibody alone, but was abrogated when both type II NKT cells and Tregs were blocked. Importantly, adoptive transfer of type I NKT cells can restore the protection against tumor in Jα18^−/−^ mice treated with anti-CD25 antibody [51]. These results indicate that type II NKT cells might suppress tumor immunity to promote tumor progression, and type I NKT cells might inhibit the immune suppressive ability of type II NKT cells.

## Avidity and stability of TCR-glycolipids-CD1d complex dictate the differential capacities of α-GalCer and its analogues for NKT activation

**Fig. 1:**
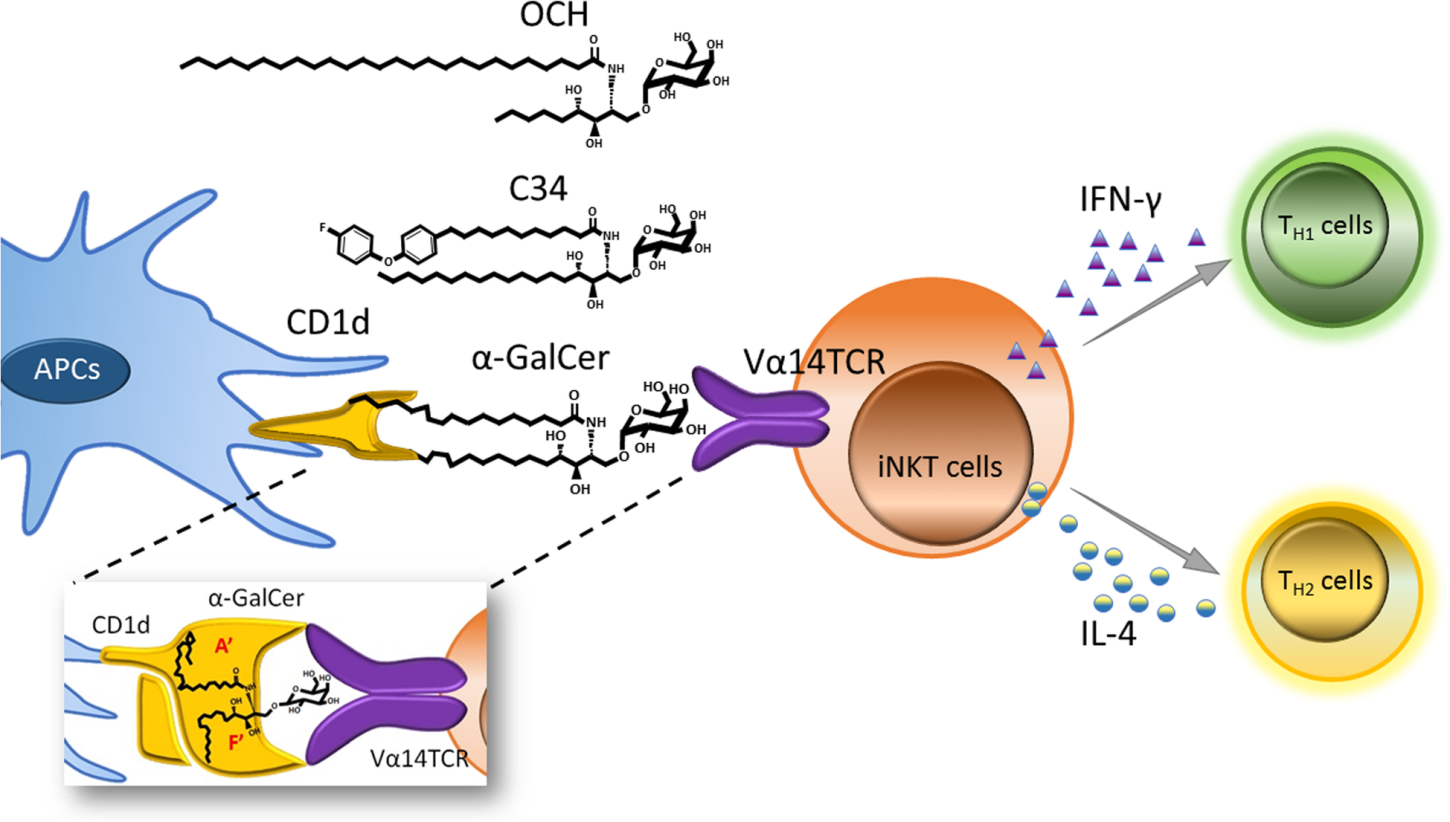
Activation of NKT cells by α-galactosylceramide and its analogs. CD1d molecule on the antigen presenting cells (APCs) presents α-galactosylceramide and various analogs, such as phenyl α-GalCer (C34) and OCH, to the Vα14 T cell receptor (TCR) of iNKT cells, and triggers the activation of iNKT cells to secrete cytokines, e.g. interferon-γ (IFN-γ) and interleukin-4 (IL-4). Modification of the α-GalCer at its acyl chain or phytosphingosine chain can manipulate the iNKT cells to produce different amount of IL-4 or IFN-γ to polarize immune response toward Th1 or Th2

α-GalCer, also known as KRN7000, is a simplified glycolipid analogue of agelasphin, which was originally isolated from a marine sponge *Agelas mauritianus* [52, 53]. α-GalCer is composed of an α-linked galactose, a phytosphingosine and an acyl chain. Preclinical evidence of the antitumor activity of α-GalCer has spurred research toward the identification of its mechanism of action [23, 54]. It is a well characterized antigen for CD1d-reactive iNKT cells in mouse and human [55–57]. X-ray crystallographic analysis of binary complex of α-GalCer and CD1d molecule revealed that the long lipid chain of α-GalCer is stabilized by hydrophobic interactions with amino acids from the β-sheet floor and helices of CD1d. Specifically, the A’ and the F’ channels of CD1d can accommodate an alkyl chain up to 26 and 18 carbon atoms long, respectively [26]. Furthermore, the length of lipids of α-GalCer can modulate the affinity of iNKT cell TCR and the threshold of iNKT cell activation [58]. OCH, an α-GalCer analogue with a shorter phytosphingosine chain, stimulates iNKT cells to secrete higher amounts of IL-4 than IFN-γ, triggering the immune response toward Th2 (Fig. 1). The possible molecular mechanisms of OCH-induced Th2 response might be related to its less avidity and stability in binding to CD1d than α-GalCer, leading to a less sustained TCR stimulation on iNKT cells [8, 59, 60]. Other α-GalCer analogues containing sulfonamide linkage to acyl chain induced Th2 response comparable to OCH in mouse splenocytes [61]. Besides, our group has shown that α-GalCer analogues containing a phenyl group in their acyl tail are more effective than α-GalCer in inducing Th1 cytokines/chemokines and human NKT cell expansion. Similar to α-GalCer, phenyl glycolipids cannot induce cytokine production in CD1d knockout mouse, suggesting that presentation of phenyl glycolipids by CD1d protein is necessary to activate iNKT cells [60]. Moreover, one of the phenyl glycolipids, 7DW8-5 which has a shorter fatty acyl chain with a fluorinated benzene ring at the end, displayed adjuvant activity for malaria vaccine and enhanced CD8^+^ T cell response in non-human primate [62]. The differential cytokine response induced by phenyl glycolipids did not result from differential usage of TCR β chain. In fact, the major β chain used by iNKT cells for the recognition of phenyl glycolipids and α-GalCer were Vβ8.1 and Vβ8.2 in mouse and Vβ11 in human. On the other hand, phenyl glycolipids displayed greater binding strengths between CD1d-glycolipid complex and iNKT cells than α-GalCer. Additionally, the rate of dissociation of CD1d-phenyl glycolipid complex from iNKT TCR was significantly slower than that of CD1d-α-GalCer complex. These results suggest that both the avidity and stability of the ternary complex of CD1d-glycolipid-iNKT TCR play a key role in dictating the induction of cytokines/chemokines. Notably, the binding strength of the ternary structure CD1d-glycolipid-iNKT TCR is much more relevant to iNKT cell activation than that of the binary structure CD1d-glycolipid, as reflected by the differential cytokine responses to glycolipids with identical lipid tails but different glycan head [63]. More importantly, unlike α-GalCer, these phenyl glycolipids do not induce NKT cell anergy nor accumulation of myeloid-derived suppressor cells (MDSCs) [10, 11, 64], which will be elaborated in later section.

In addition, the orientation and position of hydroxyl group at the galactose ring of α-GalCer are believed to be crucial for iNKT cell recognition [6]. The 2′, 3′, and 4′-OH of the galactose moiety form hydrogen bonds with Gly96a, Phe29a and Ser30a, respectively, of the invariant TCR α-chain. Upon removal of the 2′-OH, the cytokine response declined. However, the 3′- or 4′-deoxy or -fluoro analogues of α-GalCer remain active [65]. According to the crystal structure of ternary complex of NKT TCR/α-GalCer/CD1d, the 6′-OH of the galactose moiety of α-GalCer points toward solvent [6], and thus it might or might not influence the binding of iNKT TCR to α-GalCer/CD1d complex. Indeed, addition of an extra Gal [66] or small fluorophores [67] at 6′-OH of the galactose moiety retains the activity to stimulate NKT cells. Conjugation with polyethylene glycol at 6′-amide group of the galactose moiety activates murine iNKT cells more efficiently than α-GalCer. When acting as an adjuvant for the β-galactosidase protein vaccine, the α-GalCer analogue with pegylation, which increased the water solubility, at the 6′-amide of galactose moiety elicited high titers of antigen-specific antibodies in mouse, even though it induced lower production of IFN-γ than α-GalCer [68]. In contrast, α-GalCer analogue with a naphthylurea at 6′-amide of galactose moiety induced Th1 bias immune response and prevented lung metastasis of melanoma [69] whereas α-GalCer analogue with a methyl group at 6′-OH of galactose moiety induced slightly higher production of IL-4 and IFN-γ in mouse [70]. We also showed that Gal-6′- phenylacetamide-substituted α-GalCer analogues carrying p-nitro-, p-tert-butyl, or o-, m-, or p-methyl groups elicited higher IFN-γ/IL-4 secretion ratios than α-GalCer [71]. In contrast, we have recently shown that adding acyl chain at the 6′-OH of galactose moiety of α-GalCer can stimulate iNKT cells to secrete more Th2-biased cytokines [72]. Interestingly, two 6′-OH analogues of α-GalCer, NU-α-GalCer and PyrC-α-GalCer, induced strong Th1-biased immune response leading to reduced lung metastasis in the B16 melanoma model [73]. These findings suggest that modifications at 6′-OH of galactose moiety may change the interaction between NKT TCR and α-GalCer-CD1d complex, thereby modulating the cytokine secretion of iNKT cells in vitro and in vivo.

Based on the X-ray crystallography study, the NH group of the amide of α-GalCer phytosphingosine chain forms hydrogen bond to Thr156 at the α2 loop of mouse CD1d [29], indicating that the NH group might play a role in the activity of α-GalCer. Indeed, the NH group isomer, having the inverted NH stereochemistry, showed weaker activity for stimulating proliferation of mouse iNKT cells and no activity for human iNKT cells [74]. RCAI-18, α-GalCer analogue with azetidine ring, induced slightly lower levels of IFN-γ, IL-2, IL-4, IL-10 and IL-13 when compared with α-GalCer. However, RCAI-8, a RCAI-18 analogue with replacement of the azetidine ring with pyrrolidine ring, did not stimulate murine iNKT cells to produce cytokines [75]. Furthermore, Shiozaki et al. showed that replacing the amide group of α-GalCer with an ester reduced the secretion of IFN-γ and IL-4 [76]. In our study, we found that substituting the hydrogen of amide group of α-GalCer with methyl group lowered its capacity in inducing cytokine production of iNKT cell [72]. Besides, α-GalCer analogue DB06-1, which contains the substitution of a carbonyl oxygen with a sulfur atom, can increase IFN-γ and IL-2p70 production and activate NK cells in vivo [77]. In addition, α-C-GalCer, a C-glycoside of α-GalCer, displayed a more potent antimalarial activity and antitumor activity in mice [9], but it did not effectively stimulate human iNKT cells in vitro [78]. Those data suggest that different modifications of phytosphingosine or acyl chain may affect the binding affinity of TCR with glycolipids-CD1d complex, and thereby modulating the magnitude and the Th1/Th2 polarity of responses of iNKT cells.

## Clinical trials of α-galactosylceramide

In preclinical studies, the anti-cancer activity of α-GalCer has been demonstrated in tumor-bearing mouse models. Prompted by these findings, Giaccone et al. conducted a phase I clinical trial of α-GalCer in patients (*n* = 24) with advance cancer. The dose was escalated from 50 to 4800 μg/m^2^ at a schedule of intravenous injection on days 1, 8 and 15 of a 4-weekly cycle. Toxicities of α-GalCer were minimal, including vomiting, malaise and chills in one patient and grade 3 fever and headache in another patient. All side effects occurred after the first administration only. Neither drug accumulation nor serum saturation of α-GalCer was observed by pharmacokinetic analyses. Furthermore, no detectable trace of α-GalCer was found in the urine at any dose level. Surprisingly, in patients with high number of NKT cells (>333 cells/mL), the number of circulating iNKT cells rapidly declined to undetectable levels within 24 h after the first injection of α-GalCer. Even after two additional injections, circulating iNKT cells remained negligible or undetectable. Notably, patients with pretreatment iNKT cell numbers higher than median number in all patients had greater cytokine productions induced by α-GalCer. Five of 10 patients in NKT-high group showed significant increases in serum levels of both GM-CSF and TNF-α. In contrast, GM-CSF and TNF-α levels barely rose in the NKT-low group after α-GalCer administration [79]. In addition, the impacts of age and cancer status were assessed to provide information for iNKT cell-based immunotherapy [80]. The percentages of circulating iNKT cell were significantly lower in patients with melanoma (*n* = 17) and breast cancer (*n* = 10) than in healthy donors (*n* = 40). The percentages of circulating iNKT cells were also lower in patients with colorectal cancer (*n* = 33), lung cancer (*n* = 8) and RCC (*n* = 10) than healthy controls but the difference did not reach statistical significance. In addition, in vitro expansion capability of iNKT cells from cancer patient in response to α-GalCer was less than that from healthy donor. Moreover, the percentage and absolute number of circulating iNKT cells decreased with increasing age, although age did not affect the expansion capability of iNKT cells stimulated with α-GalCer [80]. These results suggest that the cytokine production and iNKT cell expansion induced by α-GalCer in patients depend on the pre-treatment circulating iNKT cell population size which is influenced by type of cancer and patient age.

Several studies have shown that α-GalCer-pulsed DC can inhibit tumor growth in mouse and expand human iNKT cells in vitro [81–83]. These findings prompted a few clinical trials to evaluate the antitumor efficacy of α-GalCer-pulsed DC. In the phase I trial conducted by Nieda et al., patients (*n* = 12) received 5 × 10^6^ α-GalCer-pulsed DC on day 0 and 14. Increased serum level of IFN-γ and number of NKT cells were observed initially but the number of NKT cells fell by day 2 after treatment. The majority of patients experienced temporary exacerbation of tumor symptoms, including enlargement of tumor, bone pain and biochemical abnormalities. However, a few patients showed tumor responses, such as decreased serum tumor markers (*n* = 2), increased necrosis in tumor (*n* = 1) and improvements in hepatocellular enzyme levels (*n* = 2) [84]. Chang et al. demonstrated that injection of unpulsed DC at week 0 and α-GalCer-pulsed matured DC at week 4 and 8 led to a dramatic expansion of NKT cells in all patients (*n* = 5) after the third injection [85]. In addition, Uchida et al. administered α-GalCer-pulsed DC into the nasal submucosa of patients (*n* = 9) and found that the number of circulating NKT cells increased [86]. Kunii et al. treated 8 patients with recurrent head and neck squamous cell carcinoma by intra-arterial infusion of ex vivo expanded autologous iNKT cells in combination with nasal submucosal injection of α-GalCer-pulsed DC. They found that both the number of circulating iNKT cells and IFN-γ-producing cells increased (7/8), However, mild to severe adverse events were observed i.e. a grade 3 pharyngocutaneous fistula (*n* = 1), low-grade fever (*n* = 4), headache (*n* = 1), and fatigue (*n* = 2). In spite of these adverse events, three partial responses, four stable diseases and one progressive disease were observed [87]. Furthermore, the administration routes of α-GalCer-pulsed DC were evaluated in patients with metastatic malignancy [88]. Increases in serum IFN-γ levels in patients were seen after intravenously injection of 5 × 10^6^ of α-GalCer-pulsed DC but were not observed in patients after intradermally injection of the same dose of α-GalCer-pulsed DC. Notably, six patients had stable disease, which was defined as no substantial increase in tumor masses or tumor markers, during the study period of 3 months. Taken together, these reports suggest that α-GalCer-pulsed DC might induce clinically beneficial immune responses in patients with cancer.

Moreover, the antiviral effects of α-GalCer on hepatitis viruses were evaluated. A randomized placebo-controlled phased I/II trial of α-GalCer in chronic hepatitis C virus (HCV) infection was conducted by Veldt et al. A total of 40 patients were enrolled and three dosage levels of α-GalCer (0.1, 1 and 10 μg/kg) were tested. α-GalCer was well tolerated in patients with HCV, with only mild adverse events including fatigue, myalgia, back pain, headache, rhinitis, fever, chills and dizziness. As shown in Giaccone’s report [79], the number of circulating iNKT dropped immediately after the first injection of α-GalCer in patients with HCV, but recovered approximately 2 days later. Subsequent second and third injection of α-GalCer did not further enhance the iNKT cell number. In several individuals, productions of IFN-γ and TNF-α were observed. One patient showed a mark decrease in HCV RNA after the first injection of α-GalCer, but no further reduction after the second and third injection. At the end of this trial, no significant changes in HCV RNA between the two groups were noted [89]. Another randomized placebo-controlled phased I/II trial of α-GalCer in 27 patients with chronic hepatitis B virus (HBV) was conducted by Woltman et al. [90], using the same dose-schedule as Veldt’s study [89]. The adverse events were mostly flu-like syndromes except that four patients, who received ≥ 1 μg/Kg of α-GalCer, had fever and severe rigors lasting for 1 h to 2 days. This might result from relatively high iNKT cell levels in the blood of HBV patients, which had high stimulatory effects on the immune system. The number of circulating iNKT cells fluctuated, similar to the report by Veldt et al. A transient rise in serum TNF-α was observed only in patients with high pretreatment iNKT cell number. Four patients showed decrease in HBV DNA levels following the first injection of α-GalCer, but only one sustained decrease in HBV DNA levels after the second and third injection of α-GalCer. In general, the HBV DNA changes in patients injected with α-GalCer was not statistically significantly different from those in placebo group [90].

Overall, the antitumor and antiviral efficacies of α-GalCer in human are deemed too meagre to warrant further clinical trials. On the other hand, the route of injection, dose and schedule might have influenced the therapeutic efficacy of α-GalCer. Although α-GalCer did not show robust clinical efficacy in these early phase clinical trials, the use of more potent Th1-biased α-GalCer analogues containing phenyl group in lipid tail might improve the anti-tumor efficacy in human in the future.

## Possible mechanisms for the limited clinical activities of α-GalCer

The lacklustre clinical efficacy of α-GalCer in cancer and hepatitis may be attributable to α-GalCer induced liver toxicity [91], NKT cells anergy [92] and myeloid-derived suppressor cells (MDSCs) accumulation [64]. These features might have undermined the clinical efficacy of α-GalCer.

**Fig. 2:**
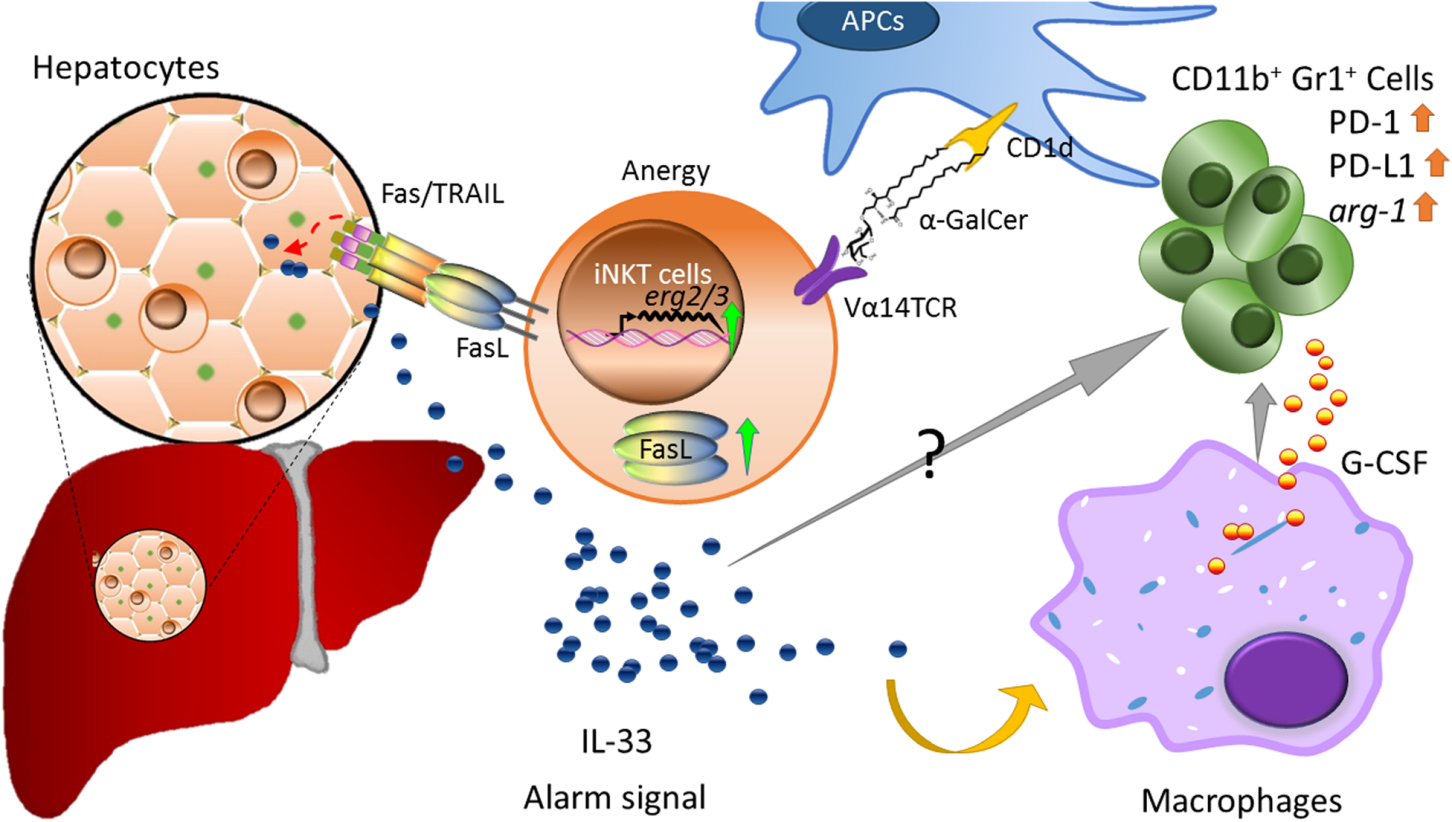
Mechanisms of α-GalCer induced anergy of iNKT and accumulation of myeloid-derived suppressor cells (MDSCs). The binding of CD1d-α-GalCer to TCR of iNKT cells triggers iNKT cell anergy via upregulation of *egr-2* or *egr-3* in NKT cells. The engagement of CD1d-α-GalCer-TCR also induces expression of FasL on iNKT cells. Binding of FasL to Fas or TRAIL on hepatocytes may cause hepatocyte injury and trigger IL-33 release, which in turns stimulated G-CSF production by macrophage, leading to increased number of MDSCs

One day after injection of α-GalCer in mice, several white spots (0.5–1 mm in diameter) were readily discernible on the surface of liver, which consisted of hepatocyte damage accompanied by lymphocyte infiltration in liver parenchyma and a rise in serum levels of SGOT and SGPT. Such hepatocyte damage induced by α-GalCer was dependent on NK/NKT cells [91]. Furthermore, we demonstrated that α-GalCer significantly upregulated the expression of TRAIL and FasL in liver iNKT cells, leading to injury of liver cells which constitutively express Fas and death receptor 5 (DR5) (Fig. 2). In comparison, negligible or only a very slight increase of the TRAIL and FasL on liver iNKT cells was detected in mice injected with the α-GalCer analogues containing phenyl group at the acyl chain [64]. Indeed, no white spots on liver were observed in mice injected with these phenyl analogues, suggesting that these phenyl glycolipids may have the advantage of little or no hepatotoxicity in human use.

Another feature of α-GalCer that may limit its clinical efficacy is that α-GalCer induces iNKT cell anergy. Upon in vitro re-stimulation with α-GalCer, splenocytes from α-GalCer-injected mice produced only low levels of cytokines and decreased proliferation ability as compared to the first injection. This unresponsive phenomenon of α-GalCer treated iNKT cells was thymus independent and can be abrogated by IL-2 [93]. It is well known that in T cells, stimulation of TCRs with weaker ligands induces anergy via up-regulation of the transcription factor egr-2/3, leading to the expression of cbl-b and programmed cell death protein 1 (PD-1) [94]. In line with T cell anergy, the α-GalCer-induced anergy of iNKT cells has been shown to up regulate the expression of cbl-b, PD1 and egr-2/3. Interestingly, α-GalCer analogues with phenyl group, which display greater binding avidity and stability to iNKT TCR than α-GalCer [60], did not induce the expression of PD-1 and cbl-b, nor anergy upon repeated treatment [64].

**Fig. 3:**
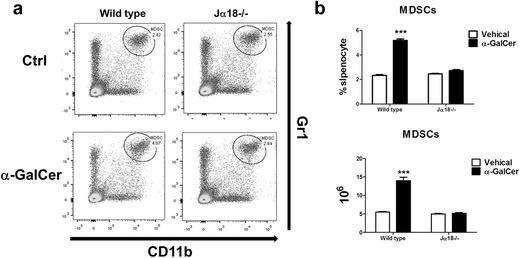
MDSC accumulation induced by α-GalCer is iNKT cell-dependent. BALB/c and Jα18^−/−^ mice (*n* = 3 per group) were intravenously injected with α-GalCer (2 μg/mouse) or vehicle (1% DMSO). Three days after administration, MDSCs (CD11b ^+^ Gr1^+^) were determined by FACS analysis. **a** Representative dot plots of MDSCs in the presence/absence of α-GalCer in wild type and Jα18^−/−^ mice were shown. **b** The percentage and total number of MDSCs in splenocyte from each mouse of indicated group are presented and shown as mean ± SD. ***, *p* < 0.01 as compared with vehicle

The lack of effective elimination of MDSCs by α-GalCer is another potential drawback. MDSC is a population of myeloid cells that co-express CD11b^+^ and Gr1^+^ surface markers and has been shown to suppress anti-CD3/anti-28 induced T cells proliferation [95], downregulate CD3ζ-chain expression [96], inhibit the CD8 T cells cytotoxicity, induce T cells apoptosis [97] and reduce the cytotoxicity of NK cells and activation of NKT cells [98]. Thus, it contributes to hyporesponsiveness of various immune effector cells, resulting in enhanced tumor progression and metastasis [99, 100]. The suppressive activities of MDSCs are achieved by regulation of L-arginine metabolic pathways. L-arginine is metabolized by arginase to generate urea and L-ornithine or is converted into citrulline and nitric oxide (NO) by inducible nitric oxide synthase (iNOS) [101]. It has been reported that MDSCs produce NO to suppress the immune response in the tumor microenvironment [102]. Repeated injection of α-GalCer maintained high level of MDSCs in the spleen and enhanced the expression of PD-1 and PD-L1 as well as arginase 1 and iNOS on MDSCs. Moreover, accumulation of MDSCs was at least in part attributed to up-regulation of G-CSF through IL-33, which was triggered by liver damage [64]. Since treatment of Jα18^−/−^ mice with α-GalCer failed to show accumulation of MDSCs (Fig. 3), in contrast to wild type mice, accumulation of MDSC induced by α-GalCer is iNKT cell-dependent. Thus, strategies to diminish the number or suppressive activity of MDSCs induced by α-GalCer might enhance the anti-tumor effect of α-GalCer. Recent report that suppression of iNOS by L-NAME, which is an inhibitor for iNOS, enhanced the anti-tumor effect of α-GalCer is consistent with this notion [103]. Another strategy is to modify the structure of α-GalCer to reduce the MDSC-promoting activity thereby enhancing its anti-tumor activity. Indeed, phenyl glycolipids did not induce MDSC accumulation in the spleen nor in the tumor microenvironment in contrast to α-GalCer. Such structural modification of α-GalCer might represent a step in the right direction for the development of more potent NKT-stimulatory glycolipids for cancer therapy [64].

## Conclusion

Various modifications on α-GalCer have been made, and studies of these α-GalCer analogues have shed light on the direction to manipulate the activation of iNKT with desired immune responses. Some of these α-GalCer analogues have demonstrated improved efficacy as anticancer agents, vaccine adjuvants and anti-autoimmune agents in animal studies. Therefore, understanding the correlation between structure and activity of α-GalCer analogues on the activation of iNKT cells and their molecular mechanism related to immune modulation should facilitate the development of potent immune modulating glycolipids for various diseases. Besides, the route of injection, dose and treatments schedule might influence the therapeutic efficacy of α-GalCer. Although the α-GalCer did not show good clinical responses in the phase I clinical trials, using low immune suppressive α-GalCer analogues and optimized treatment schedule might show better anti-tumor efficacy in human in the future.

## Abbreviations

CIA: Collagen-induced arthritis
DCs: Dendritic cells
DR5: Death receptor 5
EAE: Experimental autoimmune encephalomyelitis
GVHD: Graft-versus-host disease
HBV: Hepatitis B virus
HCV: Hepatitis C virus
IFN-γ: Interferon-γ
iGB3: isoglobotrihexosylceramide
IL-4: Interleukin-4
iNKT: invariant NKT
iNOS: inducible nitric oxide synthase
MDSCs: Myeloid-derived suppressor cells
MHC: Major histocompatibility complex
NK: Natural killer
NKT: Natural killer T
NO: Nitric oxide
PD-1: Programmed cell death protein 1
TCR: T cell receptor
Treg: Regulatory T
α-GalCer: α-galactosylceramide
